# Microbiome-Induced Microenvironmental Changes Before and After Breast Cancer Treatment

**DOI:** 10.3390/microorganisms13051057

**Published:** 2025-05-01

**Authors:** Jeongshin An, Hyungju Kwon, Young Ju Kim, Byung-In Moon

**Affiliations:** 1 Institute of Convergence Medicine Research, Ewha Womans University Mokdong Hospital, Ewha Womans University College of Medicine, Seoul 07985, Republic of Korea; 2 Department of Surgery, Ewha Womans University Mokdong Hospital, Ewha Womans University College of Medicine, Seoul 07985, Republic of Korea; hkwon@ewha.ac.kr; 3 Department of Obstetrics and Gynecology, Ewha Medical Institute and College of Medicine, Ewha Womans University, Seoul 07804, Republic of Korea; kkyj@ewha.ac.kr

**Keywords:** breast cancer, microbiome, cancer treatment, neutropenia, anemia

## Abstract

Breast cancer treatment, including surgery, chemotherapy, radiotherapy, and endocrine therapy, can affect the microbiome and microenvironment of the human body. The present study examined how the microbiome changes before and after treatment in patients with breast cancer and explored variations in the expression of putative proteins linked to these shifts. Forty-five patients enrolled in this study, and blood samples were collected and sequenced to analyze microbiome composition. Using these sequences, we estimated and compared the putative protein expression levels. In addition, complete blood count results were analyzed to evaluate treatment-induced alterations. The findings indicate that treatment leads to microbiome modifications associated with changes in the human microenvironment. Moreover, key putative proteins involved in these processes were identified. This study provides valuable insights into how breast cancer treatment affects the microbiome and helps elucidate the potential role of microbial protein expression in patient outcomes.

## 1. Introduction

Breast cancer is the most common cancer in women worldwide, and despite advances in early diagnosis and treatment, it remains a major cause of female cancer-related deaths [1]. The gut microbiota play a crucial role in regulating metabolic processes in the host [2]. Similarly, it can be assumed that changes in human symbiotic bacteria are associated with surgery, chemotherapy, radiation therapy, and endocrine therapy, which can influence human metabolism, and lead to differences in the host microbiome and microenvironment. Approximately two-thirds of the 271 drugs examined in that study are reportedly metabolized by at least one bacterial strain [3]. This indicates that individual variations in drug responses may arise, even when the same drug is administered. Understanding the changes in the microenvironment within the human microbiome host relationship before and after treatment could facilitate the development of more effective therapeutic strategies.

When breast cancer is diagnosed, surgery, chemotherapy, radiation therapy, and endocrine therapy are administered, and patients experience various side effects. Chemotherapy-induced neutropenia (CIN) is one of the most common side effects of chemotherapy, and the risk of infection increases [4]. These side effects, including inflammation resulting from abnormal immune responses, disrupt the gut microbiome [5]. In particular, since bacterial protein expression varies across host conditions and has been reported to influence the tumor microenvironment [6], understanding the impact of the microbiome on the human microenvironment before and after treatment is of critical importance. This study aims to demonstrate that microbial proteins can influence human physiology and subsequently alter the microenvironment, and to investigate how differences in microbial protein expression before and after treatment may contribute to these changes.

Previous studies have analyzed the effect of adjuvant or neoadjuvant therapy using anthracyclines and taxanes on the gut microbiome in patients with breast cancer. These studies indicated that the side effects of treatment, such as weight gain and neurological disturbances, may be influenced by the gut microbiome [7]. However, this study has a limitation in that it is based on stool sample analysis. The breast is a distant organ from the colon, and while microorganisms that directly interact with the colon can be clearly implicated in colorectal cancer, their association with breast cancer requires a mechanistic explanation for inter-organ communication. Although bacteria from the colon may not directly migrate to the breast, bacterial extracellular vesicles (EVs) circulating through the bloodstream or lymphatic system may exert systemic effects [8]. Therefore, in this study, we propose that bacterial EVs circulating in the blood may represent a plausible mechanism through which the microbiome influences breast cancer. In addition, most studies have not encompassed all the treatments for breast cancer, and a lack of systematic research on the dynamic changes before and after treatment exists. Therefore, a comprehensive analysis of the microbiome alterations before and after treatment is required.

In the present study, we performed a microbiome analysis of patients with breast cancer before and after breast cancer treatment. Through taxonomic and microbial functional profiling, we investigated the microbiome-mediated changes in the human microenvironment caused by breast cancer treatment and analyzed the results in conjunction with complete blood count (CBC) data.

## 2. Materials and Methods

### 2.1. Sample Collection

Serum samples were collected from 45 patients with breast cancer at Ewha Womans University Mokdong Hospital (Table 1). All the participants provided written informed consent prior to enrollment and voluntarily participated in the study. Female patients diagnosed with breast cancer were included, although those with stage IV disease were excluded. To minimize potential confounding effects on the microbiome, patients who had received antibiotics within one month prior to sample collection were also excluded. All the patients in the post-treatment group received therapy in the conventional sequence—surgery, chemotherapy, radiotherapy, and endocrine therapy—and no cases of neoadjuvant chemotherapy were included in the dataset. Samples were obtained from 36 patients before treatment following breast cancer diagnosis and from 9 patients after diagnosis and treatment. The control group was established using a matched case–control design, with four age-matched controls selected for each individual in the experimental group (1:4 matching) to minimize the potential confounding effect of age. Because the number of patients in the treated group was small, 18 samples were collected by acquiring 2 samples at different times from nine patients, with approximately three months between the 2 sampling periods. The samples from both groups were age-corrected, and the average age of the enrolled patients was 51 years. All the participants were diagnosed with stage 0–3 breast cancer, and the two groups differed in whether they were treated. The microbiota was analyzed from these serum samples, and a CBC test was performed on whole blood samples from the enrolled patients. This study was approved by the Institutional Review Board of the Ewha Womans University Mokdong Hospital (IRB No. EUMC 2014-10-005, EUMC 2020-11-028).

### 2.2. DNA Extraction and NGS Sequencing

Blood samples were collected in serum separator tubes and centrifuged at 3000 rpm for 15 min at 4 °C. Extracellular vesicles (EVs) were isolated according to previously established protocols [9]. EV-derived DNA was extracted from the serum using the DNeasy PowerSoil Kit (QIAGEN, Hilden, Germany) and quantified using the QIAxpert system (QIAGEN, Germany). The extracted DNA was then amplified using the primers 16s_V3_F (5′-TCGTCGGCAGCGTCAGATGTGTATAAGAGACAGCCTACGGGNGGCWGCAG-3′) and 16s_V4_R (5′-GTCTCGTGGGCTCGGAGATGTGTATAAGAGACAGGACTACHVGGGTATCTAATCC-3′) [10]. Library preparation and amplicon sequencing were performed according to the Illumina protocol using a MiSeq platform (MiSeq Software v4.1.0).

### 2.3. Metagenomic Analysis of Microbial EV Composition

In this study, microbiome analysis was conducted using the EzBioCloud platform to compare subgroup characteristics and perform genetic and microbial functional profiling [11]. This study was conducted using a methodology similar to that used in previous research [6]. Species-level classification was based on average nucleotide identity (ANI), with a threshold of ANI  ≥ 95% and coverage ≥ 20%. When these criteria were not met, the classification was supplemented using 16S rRNA sequences (>1300 bp). The EzBioCloud 16S rRNA database was incorporated into the QIIME 2 (version 2024.10) and Mothur (version 1.48.0) pipelines for operational taxonomic unit (OTU)-based classification by applying processes such as OTU selection, chimera sequence removal, and sequence alignment to ensure classification accuracy. This study used OTU clustering, which limits taxonomic resolution but ensures comparability with prior research. Species abundance was assessed using raw metagenomic reads. These reads were first mapped to the custom Bowtie2 database using default parameters. Following alignment, Samtools was employed to convert and sort the resulting BAM files, and Bedtools was used to calculate read coverage across core genes. To reduce the likelihood of false-positive species identification, only those reads covering at least 25% of a species’ core genes—verified by an in-house script—were retained. Species abundance was then estimated by counting the total number of reads mapped to each species and normalizing based on gene length. This approach, combined with the extensive genome database of EzBioCloud, enhanced the accuracy and reproducibility of the taxonomic profiling. In addition to taxonomic profiling, we further investigated the functional potential of the microbiome using the same metagenomic data. Microbial functional profiling was performed using the EzBioCloud platform, which applies a core gene-based metagenomic analysis pipeline. Species identification and relative abundance estimation were carried out using Kraken2 and Bowtie2, followed by read mapping to a curated core gene set to infer putative microbial functions. Microbiome data were analyzed using GraphPad Prism software (version 10.2.3), with statistical analyses restricted to data with *p* < 0.05. Linear discriminant analysis (LDA) scores were considered only for values exceeding the absolute threshold of 2. In addition, protein–protein interaction analyses and functional assessments were conducted using STRING software (version 12).

### 2.4. Statistical Analysis

Microbial differences were assessed using the Wilcoxon rank-sum test. Principal coordinate analysis (PCoA) based on the Bray–Curtis dissimilarity distance was used to cluster samples at the individual taxonomic level, ranging from phylum to species. All the analyses were conducted using Python (version 3.11.8) and GraphPad Prism (version 10.2.3). Statistical significance was set at *p* < 0.05.

## 3. Results

### 3.1. Diversity

**Figure 1:**
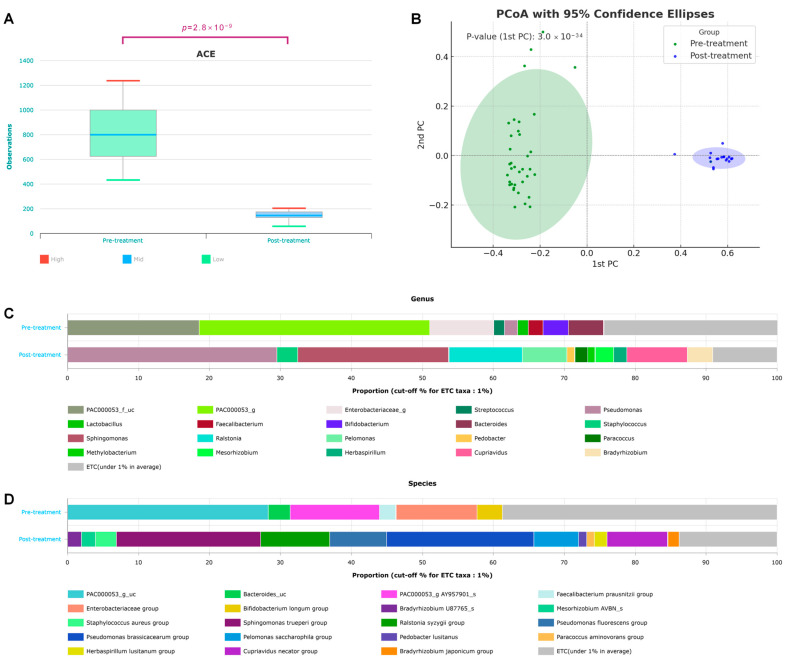
Changes in taxonomic diversity before and after breast cancer treatment. (**A**) Alpha diversity (*p* = 2.8 × 10^−9^), (**B**) beta diversity based on the Bray–Curtis dissimilarity distance (*p* = 3.0 × 10^−34^), and taxonomic composition at the (**C**) species and (**D**) genus levels in patients with breast cancer before and after treatment.

The alpha and beta diversities of the two groups before and after treatment were significant (Figure 1). The alpha diversity showed statistically significant differences for all ACE, Chao1, NP Shannon, Shannon, Simpson, and phylogenetic diversity methods (Supplementary Figure S1). In particular, the median ACE value dropped from 800 to 150 after treatment, indicating reduced microbial diversity (*p* = 2.8 × 10^−9^) (Figure 1A). Supplementary Figure S1 shows a significant decrease in phylogenetic diversity after treatment, with the post-treatment group exhibiting lower diversity and a narrower distribution. The Wilcoxon rank-sum test (*p* = 2.8 × 10^−9^) demonstrated a statistically significant reduction in microbial evolutionary diversity. Beta diversity was sufficiently different, such that the 95% confidence ellipses did not overlap before and after treatment, and *p* = 3.0 × 10^−34^ (Figure 1B). Before treatment, the genera PAC000053 and Enterobacteriaceae_g were predominant. However, after the treatment, *Pseudomonas* and *Sphingomonas* were the dominant genera (Figure 1C). At the species level, the PAC000053_g_uc and Enterobacteriaceae groups were prevalent before treatment, whereas the *Pseudomonas brassicacearum* and *Sphingomonas trueperi* groups increased after treatment (Figure 1D).

### 3.2. Taxonomic Shifts in the Microbiome Before and After Treatment

**Figure 2:**
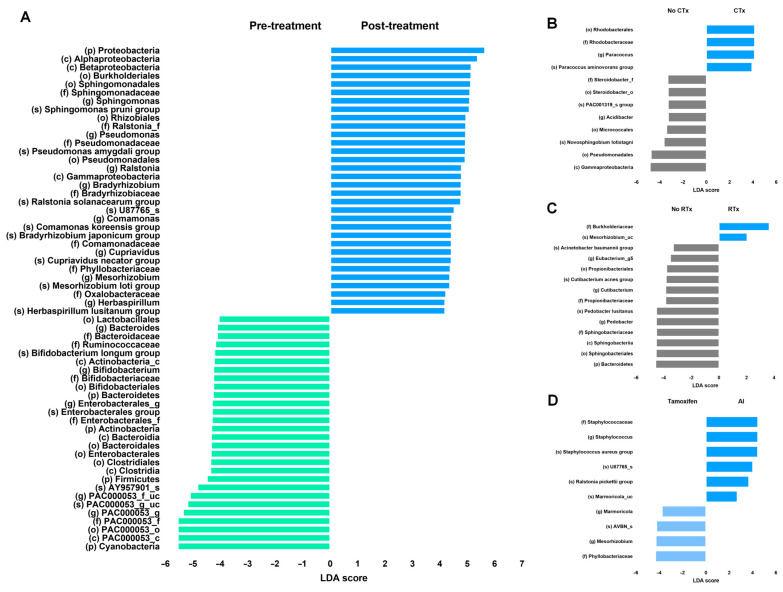
LEfSe analysis of differentially abundant taxa before and after breast cancer treatment. LEfSe analysis before and after (**A**) all breast cancer treatments, (**B**) chemotherapy, (**C**) radiation therapy and (**D**) endocrine therapy. No CTx: non-chemotherapy group, CTx: chemotherapy group, No RTx: non-radiation therapy group, RTx: radiation therapy group, tamoxifen: tamoxifen-using group, and AI: aromatase inhibitor-using group.

Linear discriminant analysis (LDA) scores were compared to evaluate the differences in the microbiota before and after breast cancer treatment (Figure 2). First, the microbiomes before and after all breast cancer treatments were compared (Figure 2A). The treatments for breast cancer include surgery, chemotherapy, radiation therapy, and endocrine therapy. Since numerous differences were observed in the microbiome before and after treatment, we performed linear discriminant analysis effect size (LEfSe) on bacteria with an LDA score of ≥4. The bacterial taxa that were relatively more abundant after treatment included the phylum Proteobacteria, classes Alphaproteobacteria and Betaproteobacteria, order Burkholderiales, and family Sphingomonadaceae. All the patients in the treatment group underwent breast cancer surgery; some received chemotherapy or radiation therapy, whereas others did not. However, all the patients in the treatment group received endocrine therapy. The comparison of the microbiome between patients who received chemotherapy and those who did not is presented in Figure 2B, with bacterial taxa having an LDA score of ≥2 highlighted. The bacteria that were more frequently observed in the chemotherapy group included the order Rhodobacterales, family Rhodobacteraceae, genus *Paracoccus*, and species *Paracoccus aminovorans*. In contrast, the non-chemotherapy group tended to show relatively greater bacterial diversity. A comparison based on the presence or absence of radiation therapy is shown in Figure 2C. Patients who underwent radiation therapy exhibited fewer bacterial taxa with high LDA scores than those who received chemotherapy. Bacterial taxa with an LDA score of ≥2 that were enriched following radiation therapy included family Burkholderiaceae and species *Mesorhizobium*_uc. All the treated patients also received endocrine therapy. To assess the effect of endocrine therapy, the patients were divided into tamoxifen and aromatase inhibitor (AI) users (Figure 2C). Based on an LDA score threshold of ≥2, family Staphylococcaceae and genus *Staphylococcus* were more frequently observed in the AI-treated group, whereas the family Phyllobacteriaceae and the genus *Mesorhizobium* were relatively more abundant in the tamoxifen-treated group. However, due to the observational nature of this study and potential confounding factors, these findings should be interpreted with caution.

**Figure 3:**
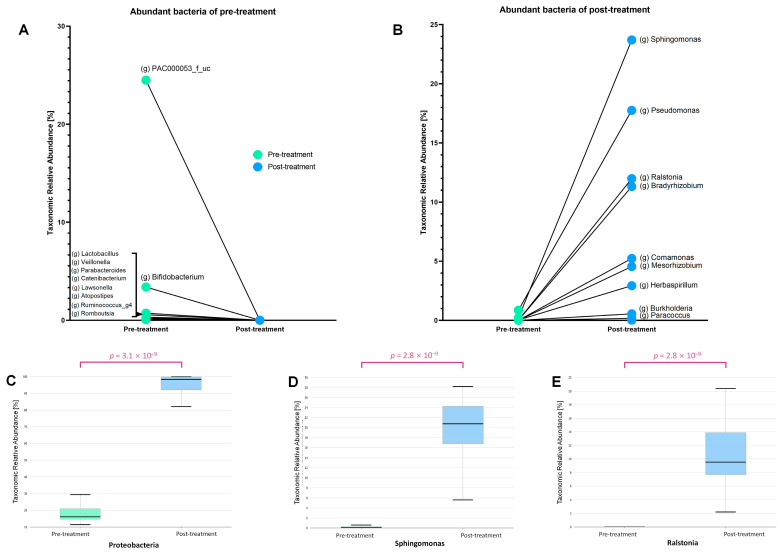
The relative abundance of several bacterial genera changed significantly before and after treatment. (**A**) Before treatment, genera such as *Lactobacillus*, *Bifidobacterium*, and *Ruminococcus* were more abundant but drastically decreased post-treatment. (**B**) After treatment, genera including *Sphingomonas*, *Pseudomonas*, *Ralstonia*, and *Bradyrhizobium* showed a significant increase. (**C**) The phylum Proteobacteria exhibited a marked rise in abundance (*p* = 3.1 × 10^−9^), with specific increases observed in (**D**) *Sphingomonas* and (**E**) *Ralstonia* (*p* = 2.8 × 10^−9^ for both).

Differences in the microbiomes before and after treatment were assessed by comparing bacterial taxa commonly present in both groups (Figure 3). The genera PAC000053_f_uc, *Bifidobacterium*, *Lactobacillus*, *Veillonella*, and *Parabacteroides* were abundant before treatment but showed a significant decline afterward (Figure 3A). In contrast, the genera *Sphingomonas*, *Pseudomonas*, *Ralstonia*, and *Bradyrhizobium* were present in low abundance before treatment, but exhibited a marked increase following treatment (*p* = 3.1 × 10^−9^) (Figure 3B). Among the bacterial taxa that increased after treatment, Proteobacteria were the most dominant (phylum *p* = 2.8 × 10^−9^) (Figure 3C). Notably, *Sphingomonas* exhibited the greatest increase among the post-treatment enriched bacteria (*p* = 2.8 × 10^−9^) (Figure 3D). Similarly, *Ralstonia*, another genus belonging to the phylum Proteobacteria, showed significant differences in abundance before and after treatment (*p* = 2.8 × 10^−9^) (Figure 3E).

We aimed to investigate the differences in the bacterial protein composition before and after treatment based on the collected data. The results below present a list of proteins with an LDA effect size of ≥3 in both groups (Table 2). A significance threshold of 0.05 was applied to both raw and FDR-adjusted *p*-values. Proteins that were predominant before treatment included Mrr protein and arginine kinase, whereas proteins that were enriched after treatment included iron-complex outer membrane receptor proteins, methyl-accepting chemotaxis proteins, and putative tricarboxylic transport membrane proteins.

**Figure 4:**
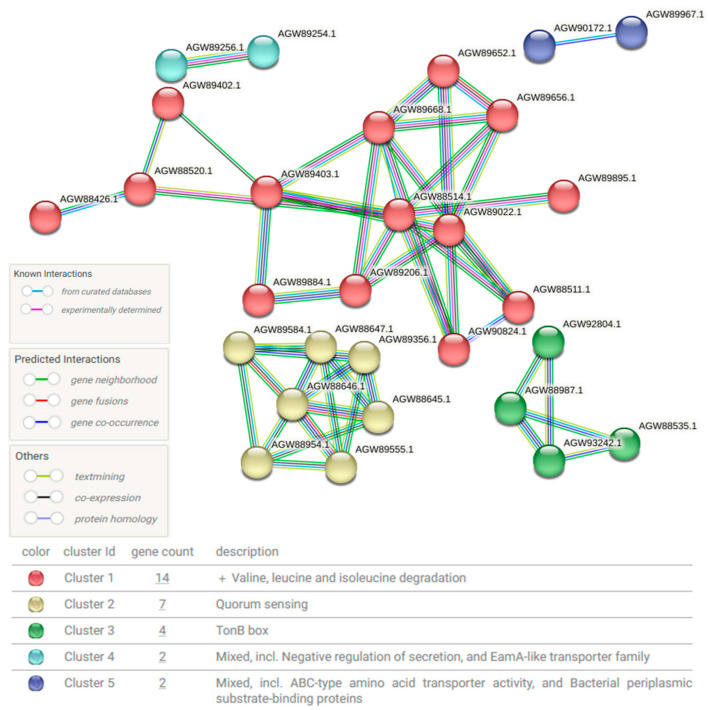
Schematic diagram of protein–protein interactions (PPIs) in *Ralstonia* after treatment. The diagram illustrates changes in PPIs based on the *Ralstonia* database following treatment. Circular nodes represent proteins classified into different functional categories, with relationships depicted in different colors to indicate known interactions, predicted interactions, and other types of associations. The network is divided into five clusters, each grouping proteins with close interactions, thus highlighting functional relationships.

Based on the increase in *Ralstonia* after treatment, microbial functional profiling using features with an LDA effect size of >2.5 was performed and classified using the STRING database (version 12.0) (Figure 4). This schematic diagram was analyzed based on the database of *Ralstonia pickettii*, one of the bacteria that increased after AI use (Figure 2D). The proteins produced by this bacterium can be categorized into five major clusters. Cluster 1 (red) represents proteins involved in valine, leucine, and isoleucine degradation. Cluster 2 (yellow) consists of proteins related to quorum sensing. Cluster 3 (green) corresponds to the TonB box. Cluster 4 (cyan) includes proteins associated with negative regulation of secretion and the EamA-like transporter family. Cluster 5 (blue) comprises proteins involved in ABC-type amino acid transporter activity and bacterial periplasmic substrate-binding proteins.

CBC analysis, including differential and platelet counts, was performed before and after treatment (Table 3). Blood samples were collected 1 week before or after microbiome sequencing to assess changes in hematological parameters. Several parameters showed statistically significant differences between the two time points. The white blood cell count and absolute neutrophil count were significantly higher before treatment, whereas the basophil count was significantly elevated after treatment. Hemoglobin levels and red cell distribution width were also significantly higher before treatment. In addition, the platelet count, plateletcrit, and platelet distribution width were significantly elevated prior to treatment.

## 4. Discussion

In this study, we aimed to investigate how systemic cancer treatment-induced microbiome alterations affect the host microenvironment of patients with breast cancer. To the best of our knowledge, few studies have investigated the microbiome changes in patients who have completed primary breast cancer treatment and are undergoing endocrine therapy. In this study, we specifically focused on how surgery, chemotherapy, radiation therapy, and endocrine therapy influence the microbiome composition and its predicted proteomic changes, linking these findings to alterations observed in blood test results.

We observed significant differences in the microbiome composition before and after treatment, as chemotherapy and radiation therapy reduced the relative abundance of symbiotic microbes, leading to notable shifts in the microbial populations (Figure 1). We observed that the bacterial count decreased and the phylogenetic diversity also exhibited a significant decline (Supplementary Figure S1). Although the levels of several bacteria were significantly reduced, some exhibited a notable increase after the treatment, which is notable because they thrived in harsh environments. Among these, *Sphingomonas trueperi* and *Pseudomonas brassicacearum* exhibited prominent increase in abundance (Figure 1). *Sphingomonas* is commonly found in soil and water and includes opportunistic pathogens [12]. Notably, previous microbiome studies have suggested a protective role of Sphingomonadaceae in breast cancer [13], and *Sphingomonas yanoikuyae* is reportedly more abundant in normal tissues than in breast cancer tissues [14]. A strain of *Pseudomonas brassicacearum* is characterized by its ability to suppress fungal pathogens, produce toxic metabolites, and form biofilms [15]. The observed increase in *Pseudomonas brassicacearum* and *Sphingomonas trueperi* may be attributed, in part, to their shared ability to form biofilms. Biofilm-forming bacteria are known to be more resistant to antibiotics and environmental stressors, which likely provided these species with a survival advantage following various therapeutic interventions [16].

The order *Rhodobacterales* was more frequently observed in the group that received chemotherapy, and both the genus *Paracoccus* and the species *Paracoccus aminovorans*, which belong to this order, were also abundantly detected. After chemotherapy, cellular damage and inflammation lead to increased tissue destruction and cell death, resulting in the release of cellular components, including amino acids, into the surrounding tissue. Given that *Paracoccus aminovorans* utilizes amino acids—particularly glycine, alanine, and valine—as its primary energy sources [17], such conditions may create a favorable environment for its growth. The Burkholderiaceae family was dominant in the RTx group that received radiation therapy (Figure 2). Among the bacteria in this family, *Mesorhizobium* was the second most characteristic taxon observed following radiation therapy. Although *Mesorhizobium* is primarily known as a symbiotic bacterium associated with plants, it has also been isolated from human blood [18]. Radiation therapy induces the production of a substantial amount of reactive oxygen species (ROS), leading to a highly oxidative microenvironment [19]. Given that *Mesorhizobium* harbors superoxide dismutase enzymes capable of detoxifying ROS [20], it is presumed to have a survival advantage under such oxidative stress conditions induced by radiation exposure. In the case of endocrine therapy, the number of patients included in each group was relatively small. Furthermore, both the tamoxifen-treated group and the AI-treated group shared similar bacterial taxa, such as *Marmoricola*. Therefore, dividing the two groups may have limited significance. Nevertheless, in the AI group, the family Staphylococcaceae, which includes the genera *Staphylococcus* and *Staphylococcus aureus*, was predominant. Since these bacteria are associated with opportunistic infections, their predominance suggests that they may have proliferated in an immunocompromised state [21]. In clinical practice, monotherapy is generally not employed for non-metastatic hormone receptor-positive breast cancer; surgery and endocrine therapy constitute essential components of standard treatment, often supplemented by chemotherapy or radiotherapy. As such, potential interaction effects among these therapies cannot be entirely excluded, and this limitation should be considered when interpreting the results of this study.

In addition to the presence or absence of microbial taxa, focusing on how shifts in symbiotic bacteria influence microbial protein expression and host physiology is crucial. Given that nearly half of all metabolites in human plasma originate from bacteria [22], it is reasonable to infer that changes in host conditions modulate microbial activity and protein expression. As shown in Table 2, the proteins that were dominant before treatment are primarily involved in bacterial defense mechanisms, particularly in the restriction of foreign DNA (e.g., Mrr protein) [23]. In contrast, the proteins that increased after treatment were predominantly membrane-associated proteins, likely involved in drug transport (e.g., ATP-binding cassette (ABC) drug transporters) and cellular responses to administered therapies (e.g., Methyl-accepting chemotaxis protein) (Table 2 and Figure 4). The overexpression of ABC transporters is known to contribute to resistance against anticancer agents by promoting drug efflux [24]. In parallel, methyl-accepting chemotaxis proteins may facilitate bacterial survival by guiding movement toward microenvironments with lower drug concentrations [25]. Such adaptive mechanisms can lead to treatment failure, recurrence of infection, and the emergence of drug-resistant strains, ultimately posing considerable health risks to the host. The upregulation of iron-complex outer membrane receptor proteins that interact with the TonB box likely reflects a bacterial adaptation to iron-restricted host conditions, as seen in chemotherapy-induced anemia [26]. Chemotherapy induces bone marrow suppression, leading to reduced red blood cell production and subsequent iron deficiency [27]. Bacteria are likely to upregulate these proteins to enhance iron acquisition, which may exacerbate iron deficiency in humans.

A notable post-treatment observation was the emergence of leukopenia and anemia as significant hematological changes (Table 3). *Ralstonia* was particularly prominent among the bacterial taxa that were consistently present but exhibited a marked post-treatment increase. Microbial functional profiling revealed that *Ralstonia* showed significant upregulation of proteins involved in the degradation of branched-chain amino acids, specifically valine, leucine, and isoleucine (Figure 3). These amino acids play crucial roles in cellular growth and energy metabolism, as well as in the biosynthesis of neutrophils [28] and erythrocytes [29]. Therefore, the upregulated *Ralstonia* proteins may have contributed to the exacerbation of anemia and neutropenia by competing with the host for critical components of the energy metabolism system. Furthermore, bacterial quorum-sensing proteins, particularly those of pathogenic bacteria, influence immune regulation [30]. Quorum-sensing molecules disrupt inflammatory responses and immune regulation by suppressing cytokine production in immune cells and impairing the function of regulatory T cells and macrophages [31]. These effects may contribute to host neutropenia and immunosuppression. The TonB box, a bacterial domain involved in iron acquisition, is of particular interest because iron is essential for hemoglobin synthesis, and disruptions in iron metabolism are directly associated with anemia. An increase in the TonB system can promote bacterial infection, deplete iron in the host, and contribute to anemia [26]. These findings suggest that while anemia in the host may be primarily induced by chemotherapy or radiation therapy, it may be further aggravated by metabolic competition with symbiotic bacteria within the host. These proteins may be actively modulated by host symbiotic bacteria that undergo significant changes in response to treatment-induced environmental pressures. The compensatory regulation of microbial proteins in response to these hematological changes further underscores the role of the microbiome in shaping the host microenvironment.

In summary, this study provides a novel perspective on the relationship between treatment-induced hematological changes, such as leukopenia and anemia, and the microbiome, thus highlighting the broad implications of microenvironmental alterations. The main limitation of this study is the small number of treated patients, which may affect the statistical power and generalizability. While two time point samples per patient increased the total sample count to 18, future research with larger, more balanced cohorts and controlled dietary variables is needed. In addition, participant heterogeneity, reliance on in silico functional predictions without experimental validation, and the observational design further limit the ability to draw causal inferences. Moreover, the V3–V4 region of the 16S rRNA gene was selected for its broad utility and genus-level resolution, though it may underrepresent certain taxa like Verrucomicrobiota or specific archaea due to primer mismatches [32]. Nonetheless, these findings may serve as a foundation for future, more robust investigations. Conventional medical research has predominantly focused on treating diseases by targeting the human body alone, whereas the influence of symbiotic bacteria on disease progression and therapeutic responses has often been overlooked. Understanding the reciprocal interactions between the host and its microbiome, where the microbiome is influenced by the host, could provide novel insights into disease mechanisms and therapeutic strategies. Further research into these host-microbiome interactions may thus pave the way for more effective and personalized treatments in oncology.

## 5. Conclusions

This study demonstrates how systemic cancer treatment reduces microbiome diversity and alters putative bacterial protein expression. The observed microbiome changes are closely linked to hematological alterations, such as leukopenia and anemia, highlighting the role of bacterial protein regulation in the human body. These findings suggest that bacterial responses to treatment may influence the human microenvironment, potentially affecting therapeutic outcomes. Recognizing the dynamic interactions between the host and its microbiome, continued research may pave the way for microbiome-targeted strategies to enhance personalized and effective cancer therapies.

## Figures and Tables

**Table 1:** Patient characteristics.

		Pre-Treatment	Post-Treatment
Number of patients (%)		36 (100%)	9 (100%)
Age (yr, mean ± SD)		51.2 ± 6.3	51.2 ± 6.1
BMI (kg/m^2^, mean ± SD)		22.5 ± 2.9	22 ± 3.0
Menopausal status	premenopausal	16 (44.4%)	5 (55.6%)
	postmenopausal	20 (55.6%)	4 (44.4%)
Estrogen receptor	positive	26 (72.2%)	9 (100%)
	negative	10 (27.8%)	0 (0%)
Progesteron receptor	positive	24 (66.7%)	9 (100%)
	negative	12 (33.3%)	0 (0%)
HER-2/new	positive	12 (33.3%)	1 (11.1%)
	negative	24 (66.7%)	8 (88.9%)
Tumor size	0~2	22 (61.1%)	1 (11.1%)
	2~5	13 (36.1%)	8 (88.9%)
	5~	1 (2.8%)	0 (0%)
Nodal status	positive	9 (25%)	2 (22.2%)
	negative	27 (75%)	6 (66.7%)
Stage	stage 0	0 (0%)	1 (11.1%)
	stage I	20 (55.6%)	2 (22.2%)
	stage II	13 (36.1%)	3 (33.3%)
	stage III	3 (8.3%)	3 (33.3%)
Chemotherapy	yes	0 (0%)	7 (77.8%)
	no	36 (100%)	2 (22.2%)
Targeted therapy	yes	0 (0%)	1 (11.1%)
	no	36 (100%)	8 (88.9%)
Radiation therapy	yes	0 (0%)	7 (77.8%)
	no	36 (100%)	2 (22.2%)
Endocrine therapy	AI	0 (0%)	6 (66.7%)
	tamoxifen	0 (0%)	3 (33.3%)

**Table 2:** Differential bacterial proteins between pre- and post-treatment.

Ortholog	Definition	Pathway	Module	LDA Effect Size	*p*-Value	*p*-Value (FDR)	Pre-Treatment	Post-Treatment
K08800	NUAK family, SNF1-like kinase			3.62	2.8 × 10^−9^	1.47 × 10^−8^	0.9	0.05
K07449	similar to archaeal holliday junction resolvase and Mrr protein			3.28	2.8 × 10^−9^	1.47 × 10^−8^	0.4	0.01
K00934	arginine kinase	ko00330		3.25	1.26 × 10^−7^	2.9 × 10^−7^	0.67	0.31
K02014	iron complex outermembrane recepter protein			3.24	3.09 × 10^−9^	1.47 × 10^−8^	0.1	0.46
K03406	methyl-accepting chemotaxis protein	ko02020, ko02030		3.19	2.8 × 10^−9^	1.47 × 10^−8^	0.06	0.37
K12446	L-arabinokinase	ko00520, ko01100		3.13	6.7 × 10^−9^	2.35 × 10^−8^	0.28	0.01
K00059	3-oxoacyl-[acyl-carrier protein] reductase	ko00061, ko00333, ko00780, ko01040, ko01100, ko01130, ko01212	M00083, M00572	3.1	2.76 × 10^−9^	1.47 × 10^−8^	0.24	0.49
K07445	putative DNA methylase			3.1	1.59 × 10^−8^	4.7 × 10^−8^	0.29	0.04
K09678	[heparan sulfate]-glucosamine 3-sulfotransferase 4			3.07	3.09 × 10^−9^	1.47 × 10^−8^	0.01	0.25
K07795	putative tricarboxylic transport membrane protein	ko02020		3.04	1.28 × 10^−8^	3.98 × 10^−8^	0.03	0.25

**Table 3:** Complete blood count with different and platelet indices.

	Pre-Teratment	Post-Treatment	*p*-Value
WBC (×10^3^/µL)	6.32	4.91	**0.0105**
ANC (×10^3^/µL)	5.07	2.7	**0.0004**
Neutrophil (%)	58.92	54.68	0.0602
Lymphocyte (%)	31.37	35.49	0.1038
Monocyte (%)	7.16	7.21	0.1368
Eosinophil (%)	2.05	1.92	0.1391
Basophil (%)	0.47	0.69	**0.0313**
Hb (g/dL)	14.56	12.46	**0.0111**
Hct (%)	41.24	37.27	**0.0142**
RDW(CV) (%)	26.29	12.67	**0.0266**
Platelet (×10^3^/µL)	252.98	215.61	**0.0021**
PCT (%)	0.7	0.21	**0.0001**
MPV (fL)	10.03	9.78	0.2492
PDW (fL)	13.68	10.34	**0.0177**

WBC: white blood cell count, ANC: absolute neutrophil count, Hb: hemoglobin, Hct: hematocrit, RDW: red cell distribution width, PCT: plateletcrit, MPV: mean platelet volume.

## Data Availability

The data supporting the findings of this study are not publicly available at this time due to intellectual property and patent-related restrictions.

## Supplementary Materials

The following supporting information can be downloaded at: https://www.mdpi.com/article/10.3390/microorganisms13051057/s1, Figure S1: Comparison of alpha diversity before and after breast cancer treatment.
