# Supplementary figures and images for: Microbiome-Induced Microenvironmental Changes Before and After Breast Cancer Treatment
Source: Microorganisms. 2025 May 1;13(5):1057. doi: 10.3390/microorganisms13051057 (PMC12113986; doi:10.3390/microorganisms13051057)

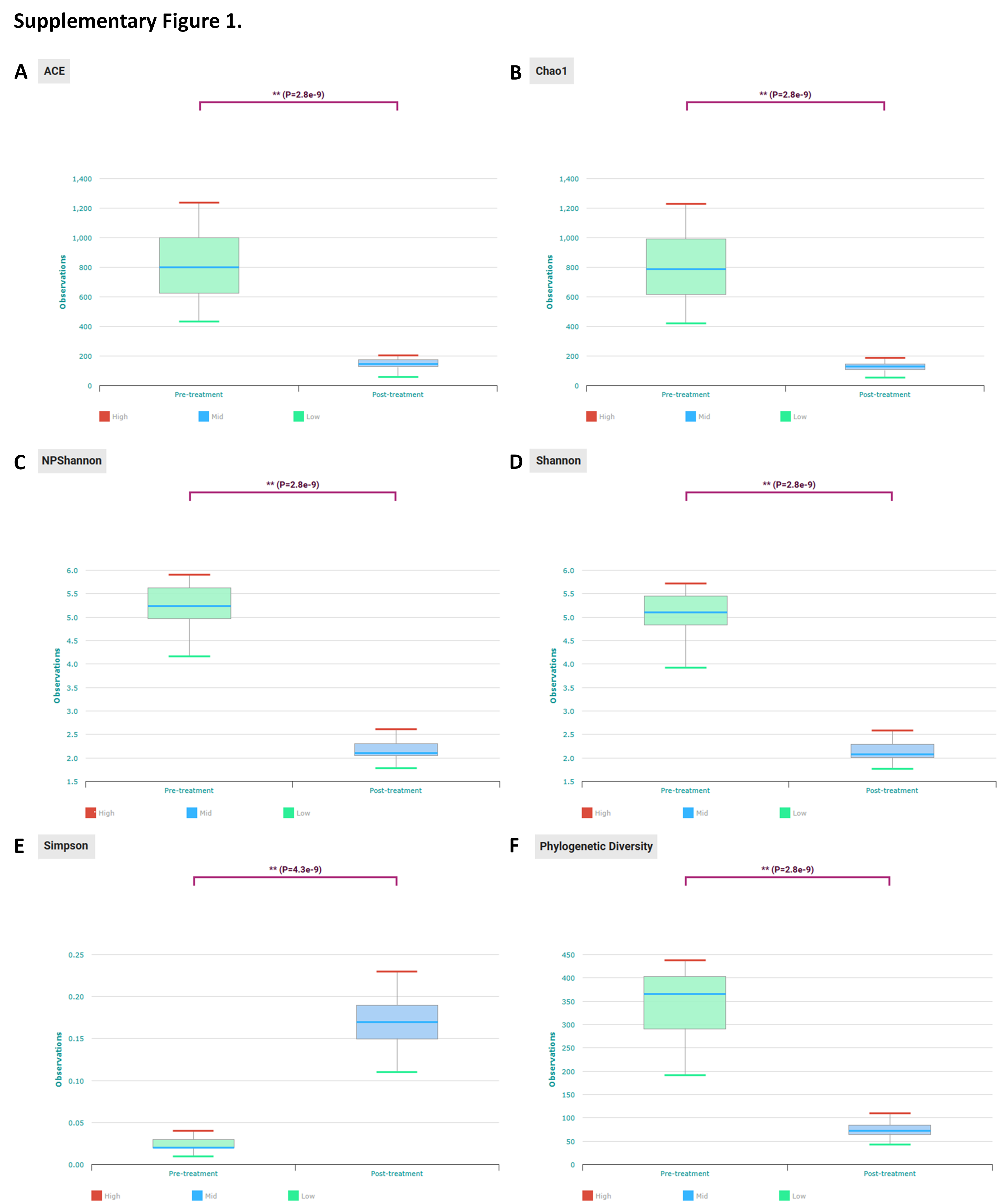

Supplement: Supplementary file 1 [file microorganisms-13-01057-s001.zip › Supplementary Figure S1 _.tif]
